# Barriers, Enablers, and Impacts of Implementing National Comprehensive Care Standards in Acute Care Hospitals: An Interview Study

**DOI:** 10.3390/nursrep15120428

**Published:** 2025-12-02

**Authors:** Beibei Xiong, Daniel X. Bailey, Christine Stirling, Paul Prudon, Melinda Martin-Khan

**Affiliations:** 1 Centre for Health Services Research, Faculty of Health, Medicine & Behavioural Sciences, The University of Queensland, Building 33, Level 2, Princess Alexandra Hospital, 199 Ipswich Road, Woolloongabba, Brisbane 4102, Australia; d23.bailey@qut.edu.au (D.X.B.); p.prudon@uq.edu.au (P.P.); m.martin-khan@exeter.ac.uk (M.M.-K.); 2 Saw Swee Hock School of Public Health, National University of Singapore, Singapore 119077, Singapore; 3 School of Nursing, University of Tasmania, Hobart 7000, Australia; christine.stirling@utas.edu.au; 4 Exeter Medical School, University of Exeter, Exeter EX1 2LU, UK; 5 School of Nursing, University of Northern British Columbia, Prince George, BC V2N 4Z9, Canada

**Keywords:** coordinated care, health policy, holistic care, implementation science, multidisciplinary care, patient-centred care, standard of care

## Abstract

**Background:** Comprehensive care is increasingly being recognised as a critical component of healthcare, with several countries endorsing it as a national standard. This study aims to explore care professionals’ perspectives on the barriers, enablers, and impacts of implementing the Comprehensive Care Standard (CCS) in acute care hospitals across Australia. **Methods:** This is a qualitative descriptive study. Participants included 28 care professionals (20 nurses, 2 doctors, and 6 allied health professionals) recruited from a broad range of Australian acute care hospitals. Data were collected using semi-structured interviews from March to August 2023. The interviews were audio-recorded, transcribed and thematically analysed. Data collection and analysis were guided by the Consolidated Framework for Implementation Research (CFIR), and implementation strategies were mapped to the Expert Recommendations for Implementing Change (ERIC). **Results:** CFIR-informed analysis identified 12 barriers and 13 enablers to CCS implementation, most prominently within the Inner Setting and Implementation Process domains. Sixteen implementation strategies were also mapped using the CFIR-ERIC Mapping Tool. The perceived impacts of the CCS implementation were multifaceted. While CCS implementation brought about changes to hospitals and improvements in patient care, it also resulted in increased workload and fatigue among staff. **Conclusions:** Enhancing CCS implementation will involve addressing the barriers and building on the enablers identified in this study. Supporting more effective implementation may help maximise the benefits of the CCS for patient care while also mitigating the increased workload and fatigue reported by staff. These findings highlight the importance of approaches that balance quality improvements with staff wellbeing.

## 1. Introduction

Patients attending acute care hospitals often have severe and multiple health needs (e.g., physical, social, and psychological needs) and require coordinated delivery of a range of health services [1]. Lack of care coordination may result in inefficient, inadequate, or fragmented care, which may cause unnecessary hospitalisation, increased length of stay, and/or adverse events [2,3]. In such circumstances, the provision of comprehensive care is crucial for addressing these diverse needs and ensuring optimal patient outcomes. Comprehensive care is defined as the “coordinated delivery of total health care required or requested by a patient” (p. 44, [4]). It involves coordinated delivery of health care through multidisciplinary collaboration, following shared decision-making with the patient, family, and/or carers [5]. Research has shown that comprehensive care programs offer many benefits, including improved patient care and health outcomes [6,7,8]. As a result, an increasing number of comprehensive care programs are being trialled or integrated into routine processes [9].

In 2017, the Australian Commission on Safety and Quality in Health Care (ACSQHC) introduced the Comprehensive Care Standard (CCS), which is one of the National Safety and Quality Health Service (NSQHS) Standards [4]. The CCS has specified four criteria and 36 actions to ensure patients receive comprehensive care [4]. Since 2019, all hospitals in Australia have been mandated to undergo an accreditation assessment against the CCS every three years [10]. To support the implementation of the CCS, the ACSQHC provides a range of resources, including an implementation guide, advisories, and training videos [9].

Despite these resources, the implementation of the CCS has encountered significant challenges [11]. An analysis of accreditation results reveals shortcomings in various actions of CCS across different criteria [12]. Identified obstacles include resource shortages, inadequate education and training, difficulties in creating a unified comprehensive care plan, and burdensome paperwork requirements [9]. ACSQHC’s survey of health organisations highlighted that developing a comprehensive care plan posed significant challenges due to the lack of a standardised care plan across disciplines and complexities in multidisciplinary care planning [13]. A survey of care professionals across Australia also highlighted the underperformance of CCS-compliant care plans. Examination of the free-text survey responses identified twelve barriers, ten enablers, and sixteen enhancement strategies for CCS implementation [14]. However, the brevity of survey responses may have limited the depth of information obtained, potentially underexploring important barriers or facilitators. A more detailed exploration could be achieved through in-depth interviews.

One study suggests positive effects of the Australian standard on patient outcomes, such as a decrease in delirium rates [15]; however, this study only focused on isolated aspects of outcomes. The survey of care professionals across Australian acute care hospitals identified that most participants believed the introduction of the CCS improved many aspects of patient care and health outcomes, including interdisciplinary collaboration, shared decision-making, care continuity, patient quality of life, length of stay, 30-day readmission, and adverse events/clinical incidents [11]. The findings also suggested heightened healthcare costs; however, the survey methodology did not allow for an in-depth probing into the participants’ perceptions of potential expense increases. Thus, there is still a need for an in-depth exploration of the impacts of a national standard for comprehensive care on patients, staff, and hospitals [9].

Internationally, several countries have adopted national standards to guide the implementation of comprehensive care, reflecting its importance in enhancing patient safety and care quality [9]. Understanding how care professionals perceive the implementation of such standards can provide valuable lessons for improving enablement and fostering more effective practices internationally.

Within this broader global context, understanding CCS implementation in acute care hospitals is particularly important. In our study, we focus on the acute care hospital setting as implementing the CCS in such a setting presents unique challenges. Acute care hospital settings are characterised by short average lengths of stay, a diverse patient population, and high patient turnover. Acute care hospitals provide a range of services, including emergency visits, surgical procedures, and outpatient clinic visits. The dynamic and fast-paced nature of acute care hospital settings requires a tailored approach to ensure the successful integration of comprehensive care practices.

Our study aimed to explore care professionals’ perspectives on the barriers, enablers, and impacts of implementing the CCS in acute care hospitals across Australia via in-depth interviews. This qualitative approach complements the prior national survey [14] by providing the contextual nuances, underlying reasons, and implementation processes that cannot be captured through quantitative methods. The research questions are

- What are the barriers and enablers encountered by care professionals in acute care hospitals during the implementation of the CCS?
- What strategies can be used to improve the implementation of the CCS?
- What are the perceived impacts of CCS implementation on patients, staff, and the hospital?

Given the increasing global emphasis on comprehensive care, our study contributes timely evidence to support ongoing national and international efforts to strengthen comprehensive care implementation.

## 2. Method

### 2.1. Design

The present study is part of a larger project exploring the implementation and impacts of the national standard for comprehensive care in Australian acute care hospitals from the perspective of care professionals, patients, and informal carers [16]. This study used a descriptive qualitative design, which remains close to the data and provides straightforward descriptions of participants’ experiences and perceptions [17,18]. This approach is particularly useful in areas where knowledge about the topic under investigation is limited [17]. From inception, we partnered with members of the eQC (evaluating Quality of Care) Patient and Carer Advisory Board [16] for their input and feedback regarding the protocol, analysis, and results by presenting and discussing the research regularly at board meetings.

### 2.2. Theoretical Framework

The study utilised the Consolidated Framework for Implementation Research (CFIR)—the Expert Recommendations for Implementing Change (ERIC) Implementation Strategy Matching Tool [19] to identify barriers, enablers, and strategies for implementation enhancement. CFIR’s domains (Intervention Characteristics, Outer Setting, Inner Setting, Characteristics of Individuals, and Process) provide a comprehensive lens to explore factors influencing implementation, enabling a structured approach for data collection and analysis [20]. In this study, the CFIR framework was used to guide the development of the interview guide and thematic data analysis and the identification of the implementation barriers and facilitators of the CCS. The ERIC was utilised to guide the choice of theoretically informed implementation strategies based on the level of agreement of expert consensus [21]. The CFIR-ERIC Matching Tool maps implementation strategies from the ERIC to CFIR constructs, guiding us in choosing strategies aligned to identified barriers and facilitators.

### 2.3. Study Setting and Recruitment

The CCS was introduced in 2017, and hospitals were required to undergo accreditation against the Standard, starting in 2019. As of the time of this study (March–August 2023), all hospitals had implemented the CCS. This study was conducted online, and participant recruitment was not restricted to any specific clinical setting or geographical region of Australia.

Recruitment of eligible participants utilised a purposive and convenience sampling approach through emails to healthcare organisations (Agency for Clinical Innovation, Australian Medical Association, Queensland Health, Health Translation Queensland, Alfred Health), clinical networks (Queensland Statewide Clinical Networks, New South Wales Statewide Clinical Networks), and social media (Facebook, X, LinkedIn, Instagram). This was supplemented using a snowballing approach with those interviewed identifying further participants.

We continued conducting interviews until data saturation in both codes and meaning was achieved, as confirmed through team discussions. Saturation was monitored through brief debriefings after each interview, during which the team reviewed emerging ideas and assessed whether additional interviews were yielding new codes or insights. It was considered achieved when no new codes were identified and no further refinements or variations in the meaning of existing themes emerged across three consecutive interviews.

Interested participants were asked to register their interest by completing an online registration form, which included providing their phone number and email address. Some participants were recruited from a prior survey study [11], where they were invited to participate in an additional interview study (reported here) following the completion of the survey. After registration, we contacted the participants via phone or email to confirm their interest and eligibility. Subsequently, we emailed them additional details about this study, which included a Participant Information and Consent sheet.

### 2.4. Inclusion and Exclusion Criteria

The inclusion criteria were care professionals who (1) reported their usual place of employment as Australian acute care hospitals, (2) had heard about the CCS (self-identified), (3) were willing to participate in an online interview, and (4) were willing to give informed consent. The exclusion criteria were less than three months of work experience in the current work organisation. The term “care professional” is a broad term that covers a wide range of professions, including doctors, nurses, and allied health professionals (e.g., physiotherapists, pharmacists, psychologists, occupational therapists, dietitians, etc.).

### 2.5. Data Collection

Interviews were conducted online via platforms such as Microsoft Teams (Version 1.6.00.1159), Zoom (Version 5.13.7), or over the phone, depending on the participants’ preference. Each interview was scheduled for an hour, following a semi-structured interview guide (Supplementary File S1). The interview guide had been collectively developed by the research team based on the CFIR framework, the CCS implementation guide, and preliminary findings of a survey study on the CCS [11]. The pool of interview questions originated from the CFIR developer’s interview questions database [22]. BX and MMK achieved consensus through a voting process to determine the relevance of the questions for selection.

### 2.6. Data Analysis

**Figure 1:**
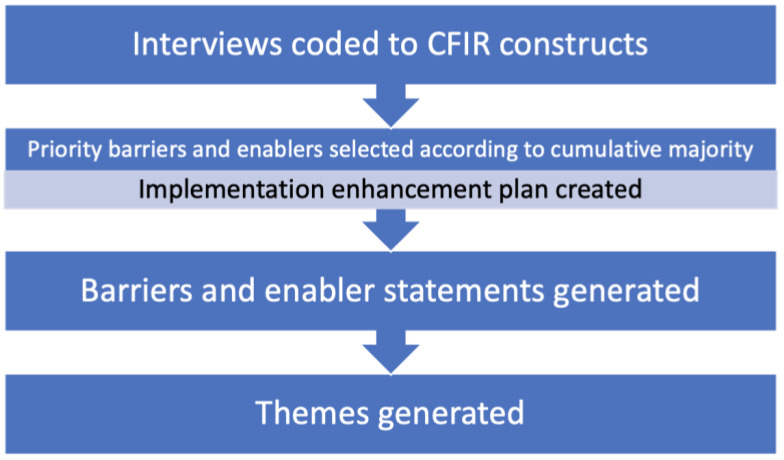
Data coding and barrier/enabler mapping process adapted from [27].

The interviews were audio recorded and transcribed verbatim by university-approved transcription services, GoTranscript (https://gotranscript.com/) and DAATS (https://daats.com.au/). These companies operate under strict confidentiality agreements, and encrypted file-transfer systems are used for upload and download. Data was analysed using NVIVO 12 software [23]. A deductive content analysis approach [24] was used to code data of barriers and enablers with predefined codes based on the CFIR. New codes were created if some parts of the texts did not directly fit any of the CFIR constructs (Supplementary File S2). While our study primarily follows a qualitative design, we used a cumulative majority approach (*n* ≥ 5) to prioritise frequently mentioned barriers and enablers for focused analysis [25]. This semi-quantitative method was applied within the broader qualitative framework to identify key areas of interest. For our dataset, the cutoff of *n* ≥ 5 produced a balanced and manageable number of prioritised barriers and enablers. Then, theme analysis [26] was used to generate barrier and enabler statements and themes using an inductive approach based on CFIR barriers and enablers. Finally, the CFIR-ERIC Matching Tool was used to create matching strategies to address prioritised barriers and amplify prioritised enablers. See Figure 1 for an overview of the analysis process.

BX performed the initial qualitative analyses, and PP reviewed and validated the analysis. Consensus discussions were held to resolve disagreements. CS, DB and MMK provided guidance and support for data analysis. All authors assisted with reviewing and refining coded themes.

The Consolidated Criteria for Reporting Qualitative Studies, as shown in Supplementary File S3 was also followed, thus adding further trustworthiness to the results.

### 2.7. Research Team and Reflexivity

The interview was conducted by a research assistant (PP; BA Hons in Psychology) and a PhD candidate (BX; BM in nursing and MSc in health sciences, with experience in qualitative research). PP led all the interviews, with support from BX through the chat function of Microsoft Teams or Zoom. PP and BX underwent two training sessions, one focusing on interview techniques and the other on the interview guide. To ensure appropriateness in terms of length and clarity, two practice interviews were conducted with senior researchers in the team (DB, MMK). The data from these practice interviews were not included in the final analysis.

A formal relationship was not established with most participants before the commencement of the study. PP and BX had prior connections with two participants—one being their colleague and the other being a personal contact of BX. These interviews followed the same semi-structured guide as all others, and notes were taken to ensure questioning remained neutral. In general, participants had limited knowledge about the interviewers (PP and BX) prior to the study. The interactions were primarily confined to phone calls and emails. Participants were informed that the interviewers represented a research team affiliated with the University of Queensland. Notably, both interviewers expressed a shared interest in the CCS, with BX concurrently pursuing her PhD on this project. Importantly, no explicit biases were disclosed by the interviewers. BX was aware of the preliminary findings of the survey study on the CCS, whereas PP was not; this difference was viewed as complementary, as PP provided a “fresh” perspective during interviews. To minimise the influence of prior assumptions, the team used field notes, cross-checking of codes and themes, and consensus discussions to ensure that interpretations were grounded in the full dataset.

## 3. Results

The average length of the interview was 52 min (SD = 5 min). Four participants opted in to review their transcripts, and none of them asked for corrections.

### 3.1. Sample Characteristics

A total of 28 interviewees participated in this study, representing five of eight Australian states and territories, with the majority from Queensland (64.3%, *n* = 18/28). All participants worked in public hospitals; the majority were female (82.1%, *n* = 23/28), with diverse backgrounds including remote/rural (10.7%, *n* = 3/28), regional (46.4%, *n* = 13/28) and metropolitan (42.9%, *n* = 12/28) settings. Nursing (71.4%, *n* = 20/28) was the dominant profession, followed by allied health (21.4%, *n* = 6/28) and doctors (7.1%, *n* = 2/28). A significant 67.9% had leadership roles (*n* = 19/28), and many had extensive experience, as nearly half had over 20 years of work experience (46.4%, *n* = 13/28). Table 1 provides a detailed overview of the demographics of each participant.

### 3.2. Barriers and Enablers

#### 3.2.1. CFIR Domains and Barrier Constructs

Thirty-five (*n* = 35) constructs from five domains were mentioned as barriers. Twelve constructs across five CFIR domains were selected for prioritisation and mapping as they represented the cumulative majority of respondents. Table 2 summarises the barrier coding results.

The Inner Setting (the setting in which the innovation is implemented) of the hospital itself emerged as a predominant barrier affecting the implementation of the CCS. Respondents identified several key elements within the inner setting, with the Availability of Resources (*n* = 21) and Structural Characteristics (*n* = 20) being dominantly mentioned. The Implementation domain is also a crucial barrier, with Doing (*n* = 13) and Planning (*n* = 12) being frequently mentioned.

#### 3.2.2. CFIR Domains and Enabler Constructs

Forty (*n* = 40) constructs from five domains were mentioned as enablers. Thirteen constructs across five CFIR domains were selected for prioritisation and mapping. Table 3 summarises the enablers coding results.

The Inner Setting of the hospital itself also emerged as a predominant enabler affecting the implementation of the CCS. Respondents most frequently cited Access to Knowledge and Information (*n* = 23), Structural Characteristics (*n* = 19), and Available Resources (*n* = 19). The Implementation domain was also a crucial enabler, with Engaging Innovation Recipients (*n* = 22) and Reflecting & Evaluating Implementation (*n* = 21) the constructs most commonly mentioned.

#### 3.2.3. Themes of Barriers and Enablers

**Figure 2:**
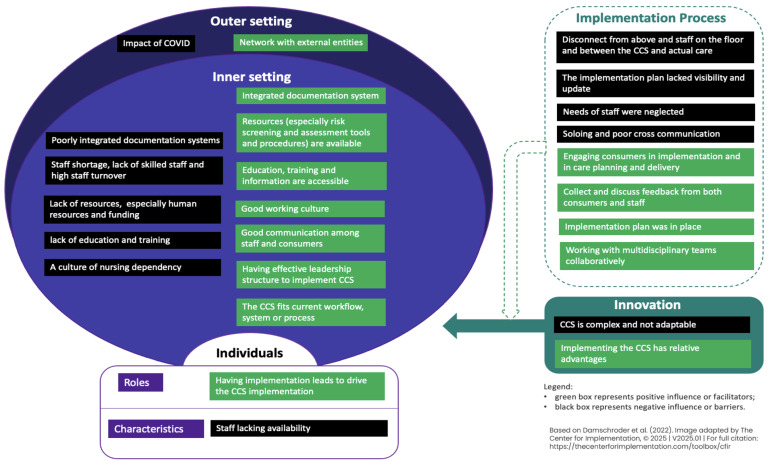
Overview of barriers and enablers on the implementation of the Comprehensive Care Standard organised according to the domains of the Consolidated Framework of Implementation Research (CFIR), adapted from [20,27].

Based on CFIR-coded barriers and enablers, barrier and enabler statements were generated. Twelve (*n* = 12) themes of barriers and 14 themes of enablers were merged from the statements, as shown in Figure 2.

#### 3.2.4. ERIC Strategy Mapping

CFIR-coded barriers and enablers were mapped to the ERIC tool to enable the selection of strategies. Strategies were selected based on the level of agreement regarding their efficacy in addressing barriers or reinforcing enablers [25]. We prioritised strategies with the highest percentage of agreement. In instances of close agreement percentages (e.g., 43% and 40% for addressing the barrier of innovation complexity), multiple strategies were selected. Following this analysis, 16 strategies surfaced as potentially effective for enhancing implementation, as outlined in Table 4. The matrix generated by the CFIR-ERIC Matching Tool is provided in the Supplementary File S4.

### 3.3. Perceived Impacts

#### 3.3.1. Perception of the Implementation

The opinions on the success of the CCS implementation varied. The feedback ranges from low confidence and scepticism to high confidence and trust in the system. Some felt that it was progressing well, although not yet complete, while others believed it was unnecessary or that the way it was being implemented was not effective.

For instance, one respondent expressed their scepticism, stating,

“I’m not confident at all in it, unfortunately. I’ve talked to a lot of nurses on the wards about it and the majority of us feel as though it was something that was actually unnecessary.”

(F07, nurse, 3–10 years of work experience)

Similarly, another respondent highlighted the incomplete nature of the implementation, saying,

“I would say that we’re still in the tweaking stages. I feel like it’s not fully there. We feel like it’s progressing, but it’s not a complete product by any structure.”

(F04, allied health, 3–10)

Conversely, some respondents expressed optimism about the progress being made. One respondent expressed hope:

“I think we’re getting there, slowly. I think we’ve still got a bit of work to do. I’m hoping that if we continue to build with some of the strategies and the ideas that we will hopefully eventually get it right.”

(F10, nurse, over 30)

Another individual stated,

“I think it will be implemented successfully. We’ve implemented a lot of things and they’ve all been done successfully. Well, they were liked by all the staff.”

(F06, nurse, 11–20)

Another respondent highlighted the importance of embracing change and incorporating CCS into broader system changes, stating,

“I think if we truly embrace these standards and we’re looking at changing-- having these big system changes that we incorporate some of this stuff into those changes so that they are right from the start, it becomes a new way of working in a way.”

(F10, nurse, over 30)

Interestingly, some participants thought care professionals’ perception of the CCS implementation is related to their level of experience, as supported by the statement:

“I suspect a lot of that will have to do with experience as well because this is just my general vibe. I find the younger staff tend to say, ‘We don’t have enough resources.’ The staff that have a great deal more experience go, ‘Oh, we’ll just get on with it’.”

(F15, nurse, over 30)

#### 3.3.2. Perceived Impacts on Hospitals

Staff reported various levels of awareness and perception of the impacts of the implementation of the CCS on hospitals. Some were not aware of any changes or reported no change, while others noted changes in documentation, policies, procedures, practices, scope, job descriptions and use of new tools. However, despite these changes, some participants expressed doubt that they led to any tangible changes in care. As one participant stated,

“I think where things were already being done well and people were getting wide broad overviews, no [chuckles], different forms, different paperwork, different policies probably not a lot of different care.”

(F03, doctor, 21–30)

Participants highlighted the challenge of recalling the timing of changes accurately:

“Timing of what happened when is always a little bit harder to recall.”

(F03, doctor, 21–30)

Regarding costs and funding, most staff members were not aware of any specific costs related to the CCS, with some believing there was no extra cost as they considered delivering care as part of routine work. While the costs associated with implementing the CCS were understandably present but difficult to quantify, others reported an increase in expenses due to the new staff or time needed for education, audits, and supporting implementation as well as costs related to printing and software licensing. As one participant stated,

“I don’t believe there are any direct costs incurred where the only costs incurred in these would be attributable to staff time, in their time from an auditing, education and supporting implementation perspective where it requires an update to forms, templates, etc. There is a time commitment and a printing cost where we change things … when you update audit tools, … there is a licensing cost … We have one at our hospital whose sole role is supporting the implementation of the comprehensive care standard. … there is one role that you have an attributed cost.”

(M01, nurse, 21–30)

This was supported by another participant,

“I manage a cost centre and 15 staff, but I would say no tangible impacts to the cost centre that I manage, …, with new physicians who were brought on to help support the comprehensive care standard implementation. … That would’ve had a financial impact…”

(F16, allied health, 3–10)

New screening and assessment tools were implemented to support the CCS implementation. For example, one participant stated,

“We have a large comprehensive care admission document, and then we’re mainly responsible for completing that, doing a lot of the screening on admission and things like that. There are a few different assessments, a few more involved cognitive assessments than previously.”

(F07, nurse, 3–10)

Another example is

“New clinical forms clinical risk screening and assessment, care planning, lots of work happening in that space.”

(F18, nurse, 11–20)

The documentation system also saw various levels of changes, with some experiencing no change, while others noted more assessments or documentation, new templates, or a shift from a paper-based to a digital system, with the implementation of new digital systems like Medtask, Cerna, Smart Referrals, and Patient Flow Manager. As stated by participants:

“Certainly yes [change in electronic systems], in terms of the templates within the patient electronic medical record…”

(F04, allied health, 3–10)

“We had Medtask implemented probably here 18 months ago, which is more of a centralised electronic communication which definitely improved communication between particularly nurses and doctors.”

(F07, nurse, 3–10)

Processes and flows were also variously affected. Some reported no changes, while others noted changes in admission assessments, handover processes, clinical pathways, and discharge planning. For example, one participant stated,

“…a lot of statewide changes, so things being driven by the state to keep things uniform across all of Queensland. We’re talking about fall pathways, pressure injury pathways, … restrictive practices … probably more formalised referral processes. They have all changed since the standard.”

(F18, nurse, 11–20)

Policies and protocols were updated to reflect the CCS, such as changes in assessment protocols and review procedures. For example, one participant stated,

“I think it felt like a lot of policy change, a lot of reviews of procedures, and potentially quite unit-specific for me without necessarily a lot of on-the-ground change. I think it’s got potential and there are processes and areas that certainly I think, that did get some change.”

(F03, doctor, 21–30)

Practices also saw some changes, including modifications to risk screening and documentation. For example, one participant stated,

“There’s a little bit more focus on cognitive screening and documentation on admission. There are plans for us to review the admission data collection for patients when they arrive at the ward and how that can be streamlined a little bit more so that people aren’t being asked the same questions in three different forms and how that can be passed around teams a little bit better both for the patients and the staff.”

(F03, doctor, 21–30)

The scope and priority of certain practices saw alterations, with some reporting no change, while others noted more focus on triaging patients, discharge planning, using interpreters, end-of-life care, and focusing on vulnerable populations. As one participant stated,

“It is very much encouraged now that if there is a situation where a patient needs to be flowed out or needs any kind of higher care than sort of our average working day, any of that is to be encouraged to be highlighted so that staffing and backup services can be supported in a more connective manner.”

(F01, nurse, 21–30)

Additionally, there were reports of expanded scopes of practice for certain practitioners, such as students. For example, one participant stated,

“In terms of students, I think there has been a push to meet workforce demands to include medical students and students of nursing to be a representative of the workforce now, there is a big push for that. We have only implemented the student in nursing, assistant in the nursing category, which is now an endorsed changed position title—under the supervision of a registered enrolled nurse, and registered nurses.”

(M04, nurse, over 30)

Finally, while there was generally no change in staff ratios, job descriptions were altered, resulting in increased workloads for some, especially nurses. As one participant summarised,

“Staff ratios have been the same and job descriptions have seemed to have changed significantly.”

(F05, nurse, over 30)

Overall, the implementation of the CCS led to a variety of changes across different aspects of care delivery and administration in hospitals.

#### 3.3.3. Perceived Impacts on Patients

Regarding access to health services, some participants were not aware of any change, while others perceived that implementing the CCS had increased access to health services, particularly due to new services being developed and more referrals being made. As two participants stated,

“I think there is generally improved access to services because there have been new services developed. There’s more of a focus towards the at-home, care at home, neuro-at-home models. I think those provide a really good bridge temporarily. That’s been a massive area for development throughout COVID-19”

(F04, allied health, 3–10)

“I think that because they’re more open to looking outside the box, I guess, that they’re making more referrals and getting more things in place for the patients now rather than before.”

(F06, nurse, 11–20)

One participant expressed variability among metropolitan, regional, and rural or remote settings, stating,

“Probably in the more non-regional settings. Probably, yes [change in access to health services]. I don’t think it’s changed much regionally or even remotely.”

(F09, nurse, 21–30)

The perceptions of care professionals regarding the impacts of implementing the CCS on patient care varied. Some reported no change or were not aware of any impact, while others were unsure if changes could be attributed to the CCS. However, among those who were aware, all believed that the implementation of CCS had improved patient quality of care, particularly by enhancing multidisciplinary care and promoting shared decision-making. As two participants stated,

“I think it is generally a more multidisciplinary approach in terms of preventing readmissions and getting the adequate supports in place, ready for home or discharge elsewhere. Yes, I think there’s more of a discussion based around it rather than clinicians deciding treatment and it being less siloed model”

(F04, allied health, 3–10)

“In our service, there are multiple new models of MDT [multidisciplinary teams] clinics that are coming to fruition at the moment and likely as a result of the change in standards and the push from maybe higher up in the hospital to do so.”

(F19, allied health, 11–20)

When it comes to health outcomes, their responses also varied. Some reported no change or were not aware of any changes, lacked access to data, or were unsure if changes were related to the CCS or other factors such as the COVID-19 pandemic. Some thought it was too early to see change. As one participant stated,

“I think it’s a little bit early to say. I would hope that there’s a reduction in the number of readmissions because of more comprehensive planning and holistic planning around a patient’s care.”

(F04, allied health, 3–10)

Others believed that the implementation of CCS had led to better patient outcomes, particularly in terms of better identification of individuals at risk. As one participant stated,

“I think for us to be able to prevent and identify risk a lot sooner makes a big difference and having some good processes around care and end-of-life for people outside of palliative care I think has made a difference in recognising delirium as well has made life—This is purely anecdotal on my part, but I do think all of these standards improve patient outcomes.”

(F05, nurse, over 30)

#### 3.3.4. Perceived Impacts on Staff

The majority of staff members believed that implementing the CCS had increased their workload, leading to staff burnout and fatigue, and this perception of workload increase was not related to lower experience. Participants stated,

“I definitely think that increased workload in terms of documentation has been a really big issue. It is still in a trial period, but as the trial period has progressed, these care plans have been neglected more and more from what I have seen in patients’ files.”

(F07, nurse, 3–10)

“So there definitely is, I am seeing, increasing workload demand with the increased expectation to deliver, but no kind of resourcing to match that expectation in delivery. And then, definitely, there is staff fatigue and staff burnout with change expectations.”

(F12, nurse, 21–30)

Nurses, in particular, were feeling the effects of this increased workload and fatigue, as one participant stated,

“With regards to the comprehensive care, there’s certainly more burden, as far as education and training on nursing staff and clinical staff to actually meet the comprehensive care, to be aware of all of the strategies and stuff that are put in place. I think that there is more of a burden on the nursing staff.”

(F22, nurse, 21–30)

This sentiment was echoed by doctors as well, with one noting,

“The comprehensive care has changed some of their data collection for admissions and probably would have a little bit of impact on time requirements, particularly of nursing staff.”

(F03, doctor, 21–30)

Staff felt that more time was allocated to CCS-related tasks, especially for those leading or championing its implementation. Some mentioned the challenges of “staying on top of the rate of change of NSQHS Standards advisories (NSQHS Standards advisories, issued by the ACSQHC, provide formal guidance and direction on the interpretation and/or assessment of the NSQHS Standards)” (F12, nurse, 21–30) because gap analysis had to be reviewed and action plan to be revised, and communication back to various committees were needed, which also caused committee fatigue.

On the other hand, a few participants did not perceive any added burden, as they were accustomed to change due to their extensive experience. As one nurse explained,

“I’m going to say no, but that’s just because that’s what we’re used to. I don’t know. 28 years in there or there’s—we’re at about 1021 changes. As nurses, we’re fairly adaptable to all of those sorts of things.”

(F09, nurse, 21–30)

Furthermore, some staff members believed that the CCS has made their work more holistic, enhancing the overall quality of patient care. As one stated,

“When the patient arrives at the emergency, okay, I’m focusing on medical issues but also I will be looking at social issues, at mental health problems at other services or if they need any help in the community, so that by that Comprehensive Standards, … It does help a lot … that will guide me … The psychosocial medical, physical, psychosocial values, preferences. By understanding Comprehensive Care, you have all these in your mind when you talk to the patient …, making sure you’re ticking all the boxes.”

(M05, nurse, 3–10)

## 4. Discussion

### 4.1. Main Findings

This interview study highlights the barriers and enablers associated with CCS implementation, as well as the perceived impacts on patients, staff, and hospital operations. Our findings confirm the challenges of policy implementation, even when written guidelines outlining goals, strategies, and a monitoring framework are provided [28]. Our findings also confirm the various perceptions of the impacts of the CCS implementation on patients, staff, and hospital operations [11]. Staff identified various changes brought about by CCS implementation in the hospital, as well as increased workload and process fatigue. However, many lacked awareness about the impacts on patients. These qualitative findings build directly upon prior ACSQHC reports [13] and the literature on CCS [11,14] by providing deeper explanatory insight into why certain challenges exist and persist, how staff experience implementation in practice, and what strategies may improve the CCS delivery.

### 4.2. Barriers and Enablers

We identified twelve CFIR barriers and thirteen CFIR enablers affecting the implementation of a national standard for comprehensive care in acute care hospitals. Most of the barriers and facilitators were already identified in the previous survey study [14]. This interview study identified additional barriers including the Complexity and Adaptability of the CCS and Assessing Needs and additional facilitators including Relative Advantage of the CCS, Partnership and Connection, Compatibility, Planning and Teaming. Constructs like Relative Priority, Motivation, and Capability were barriers and consumers (patients, families, and carers) as Implementation Team Members and Doing were enablers only identified in the survey study.

The slight differences in barriers and enablers identified between the survey [14] and interview results may be attributable to variations in the survey or interview questions. The specific questions drawn from the CFIR framework about participants’ perceptions of the CCS implementation were a focus during the interviews, providing a deeper insight into their perspectives, whereas the survey included general questions about the CCS implementation, with only specific questions regarding consumer involvement and care plan. Additionally, the participants’ leadership roles and working experience may have influenced their responses; 42.6% of survey respondents held leadership positions, while 67.9% of interviewees held such roles. Furthermore, a greater proportion of interview participants had over 20 years of working experience (46.4%) compared to survey participants (18.9%).

The complexity of the CCS emerged as a significant barrier during the interviews. Additionally, the adaptability, referring to how easily the CCS can be adapted, tailored and integrated into various hospital settings, was highlighted multiple times as another barrier. Participants felt the CCS was too broad, open to interpretation, and hard to implement, especially across diverse hospital settings. Research suggests that simplicity enhances effectiveness by improving user satisfaction and facilitating rapid proficiency [20]. Simplicity involves distilling sophisticated ideas and designs into simple and practical solutions, reducing complexity to make them easily implementable [29]. The CCS may benefit from some level of simplification, including more detailed instructions, particularly regarding governance, greater consistency in accreditation assessments and the provision of additional resources or case studies to support its application across various settings.

Despite the inherent challenges in CCS implementation, it offers several relative advantages, as supported by the literature review endorsed by the ACSQHC on the benefits of comprehensive care programs [6]. In addition, the CCS implementation was compatible with current hospital practices, as many hospitals or clinicians were already engaged in some form of comprehensive care, with CCS formalising these existing practices. Rogers’ Diffusion of Innovation theory outlines five perceived attributes of an innovation that influence its adoption and diffusion: relative advantage, compatibility, simplicity, observability, and trialability [30]. Our findings confirm prior literature suggesting that relative advantage, compatibility, and complexity are attributes likely to stimulate greater decision to adopt innovations in organisations [31]. Our findings highlight the need for policymakers and hospital leaders to demonstrate these attributes in the implementation of the CCS or trial programs, as this may convey the added value of the CCS over existing practices to those delivering care.

Implementing the CCS poses challenges, underscoring the importance of clear and detailed implementation planning, multidisciplinary collaboration, as well as fostering partnerships and connections with external entities. For instance, establishing a state-wide CCS committee and working groups can facilitate the sharing of resources and experiences, thereby minimising duplicate efforts and the reuse of effective implementation strategies. Additionally, partnering with external entities extends beyond collaboration with other hospitals. It involves establishing referral networks between health and social services [32], fostering community-academic partnerships [33], and gaining access to regional data warehouses or membership in integrated healthcare systems [34,35]. In the survey, the lack of collaboration within MDTs was recognised as a barrier, whereas collaborative work with MDTs emerged as an enabler during the interviews. This finding resonates with previous literature underscoring the significance of MDT involvement in delivering comprehensive care [9,12].

Assessing the needs of deliverers is often neglected but important. Often tasked with various responsibilities, care professionals’ needs can be overlooked, thereby affecting the effectiveness of the CCS implementation. Research indicates that addressing the needs of deliverers contributes to the ‘Quadruple Aim’, which involves the enhancement of deliverers’ work–life balance and well-being [36]. Staff should be consulted, their needs assessed, and tailored strategies should be developed to address their needs [37].

### 4.3. Perceived Impacts

CCS implementation brought various changes to hospitals, mostly around policy and protocols, workflow and process, scope or priority, documentation systems as well as job descriptions. As revealed in the survey results [11], more participants thought health care costs had increased after the implementation of the CCS. In the interview results, a few participants provided explanations about their perceptions of the change in costs. Some thought there was no extra cost as they saw delivering care as part of routine work. However, others reported an increase in costs due to the time needed for education, audits, and supporting implementation.

Consistent with the findings from the survey, some participants in the interviews were not aware of the impacts of the CCS on patient outcomes. They had not seen the data or the outcome reports, or were unsure if changes could be attributed to the CCS. Among those aware, perceptions ranged from no noticeable change to perceived improvements. Due to hospitals being at different stages of CCS implementation, some may not yet have had sufficient time to observe changes. Contrary to interview results, the earlier survey identified a small proportion of participants who perceived negative impacts. Because the interviews (March–August 2023) were conducted after the survey (October 2022–April 2023), and during a period when services were still affected by COVID-19, perception of CCS effectiveness may have shifted over time.

Implementing the CCS has increased workload, leading to staff burnout and fatigue. These perceived impacts were closely tied to the nursing-focused nature of CCS implementation. The findings suggest that the CCS implementation may not only encounter a culture of nursing dependency as a barrier but may also inadvertently reinforce it by placing documentation and coordination responsibilities primarily on nurses, thereby amplifying the pressures they already face. Neglecting the needs of deliverers may result in reduced adherence to CCS and poor quality of care, job dissatisfaction, early retirement, and increased costs of care [36,38,39]. The negative impacts of the CCS on staff should not be overlooked. Staff referred to the increased workload for implementation as a short-term burden, while the long-term burden included changes in job descriptions without additional staffing. These different types of burden should be considered separately, as their impacts are not equal. There should be greater support, particularly for young or less experienced nursing staff who noted greater impacts than experienced staff.

Taken together, these findings highlight the unique contribution of the qualitative interviews to complement the previous survey study [14]. While the survey quantified the prevalence of key barriers and perceived impacts, the interviews provided the underlying ‘why’ and ‘how’, including the contextual explanations, processes, and frustrations that shaped staff experiences. The qualitative data helped explain why certain impacts were felt more strongly by particular groups, clarified how implementation processes were carried out in practice, and identified additional barriers and facilitators not captured in the survey. This deeper insight strengthens the explanatory power of the body of work and demonstrates the added value of this qualitative component.

### 4.4. Strengths and Limitations

To the best of our knowledge, this is the first interview study exploring the views of care professionals about the implementation of CCS in acute care hospitals in Australia. Recruitment via organisational correspondence and social media, supplemented by snowballing sampling, yielded a diverse sample from various clinical settings and geographical locations, thereby providing rich insights for the study and enabling data saturation. The diversity and rich experiences of the study participants enhance the depth and breadth of the study’s findings. Moreover, the study stands out for its use of an implementation science framework in developing the interview guide and data analysis plan to explore the implementation of a national standard. This approach is novel, as few national standards undergo evaluation with such a framework, making this work pioneering and potentially valuable for similar national mandatory programs facing challenges in implementation.

Limitations of the study include the time constraints of the interviews, which may have prevented the researchers from addressing all the interview questions. Therefore, the frequency of the constructs mentioned in the interviews might be lower but still provides a reference point for understanding their occurrence. Nurses comprising the majority of participants is another limitation. Consequently, the findings predominantly reflect nursing perspectives on the implementation of the CCS. While these insights are valuable, given the close involvement of nurses in day-to-day delivery of comprehensive care, they may have shaped the emphasis placed on certain barriers, such as “a culture of nursing dependency” or “implementation work is nursing-focused.” Medical or allied health staff, who were underrepresented, may perceive differently in these aspects. Future studies could aim for a more balanced professional mix to capture a broader range of interprofessional viewpoints. Recruiting participants through organisational correspondence and social media might have resulted in a sample biased towards individuals who were already enthusiastic or disappointed about the CCS and its usefulness, potentially excluding care professionals with differing views. Despite this potential bias, we obtained a high proportion of care professionals who held leadership roles in the interviews. These participants, with their particular interest in the CCS, were crucial for providing in-depth information. However, their leadership perspectives may have shaped the salience of resource- and structure-related themes in the findings. Future research could purposively include more frontline, non-leadership staff to capture a wider range of ground-level experiences, such as by recruiting directly through partnerships with unit managers or professional associations. The sample was also skewed toward Queensland and public-sector hospitals, which may limit transferability to other Australian settings or private hospitals. Another limitation is that the ERIC was developed based on a previous version of the CFIR. Although the CFIR was updated in 2022, ERIC has not been updated yet, which means that certain constructs identified in the updated CFIR, such as Critical Incidents, Opportunity, and Teaming, do not currently have mapped strategies in the CFIR-ERIC Matching Tool. Lastly, the findings related to patient health outcomes and care experiences were based on the perceptions of participants. Therefore, careful attention is needed in interpreting these findings, and additional research is needed to validate the perceptions with objective measures of patient outcomes and experiences.

### 4.5. Recommendations for Further Research

There is a lack of research examining the real-world impacts of implementing national standards for comprehensive care. Future studies could utilise hospital data to thoroughly investigate the effects of CCS implementation, employing objective measures to assess its impact on various facets including patient, staff, and hospital operations. Furthermore, there is a need for research that not only evaluates the implementation process but also assesses the effectiveness of CCS implementation enhancement strategies and investigates unintended adverse outcomes on healthcare staff.

### 4.6. Implications for Policy and Practice

Our findings provide valuable insights into the barriers and enablers of implementing the CCS in Australian acute care hospitals, as well as strategies for improvement. These insights have significant implications for both policy and practice in Australia and internationally. In Australia, policymakers and hospital leaders can use these findings to refine national standards and develop more effective implementation frameworks.

Many of the strategies identified through the CFIR–ERIC Mapping Tool can be implemented at the local hospital level, including obtaining and using staff feedback, identifying and preparing champions, conducting educational meetings, strengthening team communication and coordination, and supporting routine monitoring and audit cycles. These actions can enhance communication, strengthen team functioning, and improve day-to-day implementation processes. Other strategies, such as developing formal implementation blueprints, integrating documentation systems, accessing new funding, and promoting the adaptability of the CCS, supporting network weaving and cross-hospital collaboration, require broader system-level support. These strategies are critical to enhancing care quality and safety. Our findings also emphasise the need to balance the benefits of CCS implementation with its potential impacts on staff, such as increased workload and fatigue. Addressing these challenges is crucial to sustaining effective and efficient care delivery.

Globally, countries with similar national standards or those considering their introduction can draw on these findings to inform policy adjustments and develop context-sensitive implementation guidelines. The strategies identified in this study, such as developing a formal implementation blueprint, identifying and preparing champions, obtaining and using staff feedback, and conducting educational meetings, can be adapted to improve care delivery. This study also highlights the importance of comprehensive, ongoing education tailored to the needs of various professional groups. Given that healthcare systems worldwide face similar challenges in upskilling diverse workforces, these insights can support the development of universal training modules and foster international knowledge exchange. Moreover, this study contributes to the growing body of evidence supporting comprehensive care as a global healthcare priority, highlighting the need for international collaboration in developing and refining care standards.

Overall, this study advances understanding of how the CCS is implemented in real-world hospital environments, highlighting both systemic barriers and practice-level enablers. By integrating CFIR and ERIC, it offers a theory-informed explanation of implementation challenges and a practical set of strategies for improvement. These insights add depth to existing national evidence and can guide future policy and implementation efforts.

## 5. Conclusions

This study identified additional barriers and facilitators beyond those revealed by the survey study, providing deeper insights into the challenges of implementing the CCS in acute care hospitals. Our study stressed the importance of simplifying the CCS, establishing effective implementation teams, strengthening clear and detailed implementation planning, integrating documentation systems, ensuring adequate resources and education, enhancing stakeholder engagement, and fostering partnerships and connections with external entities. The implementation of the CCS has driven various changes in hospitals, particularly in policy and protocols, workflow and process, scope or priority of practices, documentation systems, and job descriptions. While perceptions regarding its impact on patient care and outcomes varied, there is a consensus that it holds the potential to enhance the quality of care and improve patient outcomes. At the same time, increased workload and staff burnout and fatigue highlight the need for greater attention to staff wellbeing in implementation efforts. These insights have broader relevance for countries adopting or refining a standard for comprehensive care. Policymakers and healthcare leaders can draw on our findings to simplify the standard, provide sustained resource and training support, and strengthen collaborative, multidisciplinary approaches to implementation. Ensuring that implementation processes remain practical and well-supported will be critical to sustaining the benefits of comprehensive care initiatives internationally.

## Figures and Tables

**Table 1:** Demographics.

ID	State	Location	Size	Profession	Position	Years in Current Position	Years of Work Experience	Having a Leadership Role	Role Regards the CCS
F01	QLD	Rural	Small	Nurse	Senior registered nurse and midwife	3 months	21 to 30	No	Care delivery
F02	QLD	Regional	Small	Nurse	Senior nurse (maternity)	Less than 3	3 to 10	Yes	Care delivery
F03	QLD	Metro	Large	Doctor	Consultant physician/geriatrician	11 to 20	21 to 30	Yes	Both
F04	VIC	Metro	Large	Allied health	Dietitian, team leader	Less than 3	3 to 10	Yes	Care delivery
F05	QLD	Metro	Large	Nurse	Nurse educator (cancer and palliative care)	3 to 10	Over 30	Yes	Care delivery
F06	NSW	Metro	Large	Nurse	Nursing manager (pediatrics)	3 to 10	11 to 20	Yes	Both
F07	TAS	Regional	Large	Nurse	Junior nurse	6 months	3 to 10	No	Care delivery
F08	QLD	Metro	Large	Nurse	Nursing manager (palliative care)	3 to 10	Over 30	Yes	Care delivery
F09	NSW	Regional	Large	Nurse	Senior nurse, team leader (pediatrics)	3 to 10	21 to 30	Yes	Care delivery
F10	NSW	Metro	Large	Nurse	Senior nurse (pediatrics, CNC)	3 to 10	Over 30	Yes	Both
F11	QLD	Regional	Small	Nurse	Senior nurse (quality and safety, CNC)	3 to 10	11 to 20	Yes	Implementation lead
F12	QLD	Regional	Large	Nurse	Senior nurse (quality and safety, CNC)	3 to 10	21 to 30	Yes	Implementation lead
F13	QLD	Metro	Large	Allied health	Recreation officer (rehab)	Less than 3	Less than 3	No	Care delivery
F14	WA	Metro	Medium	Nurse	Senior nurse (pediatrics, cardiology liaison nurse, quality and safety)	Less than 3	11 to 20	Yes	Both
F15	QLD	Metro	Large	Nurse	Senior nurse—RADAR nurse navigator	3 to 10	Over 30	Yes	Care delivery
F16	QLD	Remote	Small	Allied health	Allied health manager	3 to 10	3 to 10	Yes	Both
F17	TAS	Regional	Large	Nurse	Junior nurse (surgical and medical)	3 months	Less than 3	No	Care delivery
F18	QLD	Regional	Medium	Nurse	Senior nurse (pediatrics, quality and safety)	Less than 3	11 to 20	Yes	Both
F19	QLD	Regional	Medium	Allied health	Allied health (podiatrist, outpatient)	3 to 10	11 to 20	No	Care delivery
F20	QLD	Regional	Large	Nurse	Senior nurse (CNC)	10 to 20	Over 30	Yes	Both
F21	QLD	Metro	Large	Doctor	Doctor (pediatrist, outpatient)	10 to 20	21 to 30	No	Care delivery
F22	QLD	Metro	Large	Nurse	Senior nurse (stoke rehab)	10 to 20	21 to 30	No	Care delivery
F23	TAS	Regional	Large	Nurse	Senior nurse (ICU)	3 to 10	3 to 10	Yes	Both
M01	QLD	Metro	Large	Nurse	Nursing manager	3 to 10	21 to 30	Yes	Implementation lead
M02	NSW	Regional	Large	Allied health	Allied health (occupational therapist)	Less than 3	3 to 10	No	Both
M03	QLD	Remote	Small	Allied health	Allied health (physiotherapist)	Less than 3	11 to 20	No	Care delivery
M04	QLD	Regional	Medium	Nurse	Nursing manager	3 to 10	Over 30	Yes	Implementation lead
M05	TAS	Regional	Large	Nurse	Senior nurse (ED)	3 to 10	3 to 10	Yes	Care delivery

Note: F: female. M: male. QLD: Queensland, VIC: Victoria, NSW: New South Wales, TAS: Tasmania, WA: Western Australia; Small: <100 beds. Medium: 100–499 beds. Large: ≥500 beds. RADAR: The Rapid Assessment of the Deteriorating Aged at Risk. CNC: Clinical Nurse Consultant.

**Table 2:** Barriers to the Implementation of the Comprehensive Care Standard coded using the Consolidated Framework for Implementation Research (CFIR).

CFIR Domain	Constructs	Barrier Statement	Exemplar Quotes (ID, Role, Years of Work Experience)
Innovation	Complexity *n* = 16	CCS is broad, open to interpretation, hard to implement	“The challenges are that, … some of the elements or requirements within the standard do seem to be very broad and far too open to interpretation.” (F12, nurse, 21–30) “That translation between the top-down and the bottom-up process, it’s that mystery spot in between that’s a bit tricky to figure out and is so heavily contextualised based on your setting.” (F14, nurse, 11–20) “I feel it’s a little bit hard to understand as beginner, as a student.” (F17, nurse, less than 3)
	Adaptability *n* = 6	CCS doesn’t fit various hospital settings	“It’s been difficult making any alterations or changes.” (F02, nurse, 3–10) “I think that the standard’s been written with the generic adult inpatient in mind, and I think groups that sit outside of that possibly aren’t your stock standard patient group. It is harder to tailor the intent of the standard to those groups.” (F18, nurse, 11–20) “A far-reaching standard probably needs to accommodate for the various settings that it’s implemented within and probably acknowledge that it loses a lot of its rigour in ways if we don’t have steps in place, knowing that it’ll have its limitations across really many settings and how well it’s proactively implemented at a very high level. And would be resource-driven as well.” (M02, allied health, 3–10)
Outer setting	Critical incidents *n* = 11	COVID-19 impact on health care system, staff, and consumers	“I feel there’s been another barrier I should add as well, that’s limitations to visitors as also as on the backdrop of COVID-19. I think that has also created more of a disconnection with family members being able to contribute to the care of their loved ones whilst they’re in hospital.” (F04, allied health, 3–10) “I think we probably started well, and then really COVID-19 consumed the next three years of healthcare in cost and time, and people’s energy.” (F08, nurse, over 30)
Inner setting	Available resources *n* = 21	Lack of resources, especially human resources	“[MyHospital] is a regional hospital, we’re always lacking the staff, resources like staff and we’re always looking for doctors.” (F02, nurse, 3–10) “I cover the whole of division of surgery and outpatients, which has basically six units that’s under it. And then there’s another CNC quality and safety for medicine and ED, which has another six units under it. So with the limited resource, it was more just two people doing the whole thing. Difficult.” (F11, nurse, 11–20) “I think when you haven’t got the manpower or the people on the ground, sometimes those important changes don’t have the impact that they probably should have.” (F19, allied health, 11–20) “On increasing pressure and demand, and then the things can flock. You can’t hold all the things together at the same time. You need more resources, more support.” (M05, nurse, 3–10)
	Structural characteristics *n* = 20 Work infrastructure *n* = 16 Information technology infrastructure *n* = 13	Documentation system (electronic, paper-based, or mixed) is not integrated Understaffing, high staff turnover, lack of experienced/skilled staff	“I feel, and I think that everyone in paper-based site feels like what the commission has asked for is almost unachievable for a paper-based site, which is a single view comprehensive, multidisciplinary care plan. Like all small hospitals like us, we don’t have Allied Health with their Monday to Friday, 9:00 to 5:00, so expecting them to fill out a Daily Patient Care Plan on a Saturday or Sunday is not possible. It’s very, very topical.” (F18, nurse, 11–20) “I have a different view and it’s come up several times where they’ve gone, “Well, this is how are we supposed to do this,” and I’m like, I don’t have the same screen that you have, so I can’t check to see what they see.” (F20, nurse, over 30) “When we did the initial training for EMR, plans of care weren’t really pushed per se and now we’ve got a disconnect between what we used to do on paper versus what we do within the electronic system” (F05, nurse, over 30) “Our staffing levels have always been, at the moment, particularly low. So it is not always easy to implement the Comprehensive Care Standard to a level that is sort of—a good level” (F02, nurse, 3–10) “We have a lot of new staff starting junior, like staff. They need to stress on these things to make people a lot comprehensive care competent. This is like an ongoing thing, like you need education, needs people working on the implementation.” (M05, nurse, 3–10)
	Culture *n* = 11	Implementation work is nursing-focused and -dependent	“We’ve got people in offices dictating how nurses work on the floor, but have no idea what it’s to be on the floor, and everything is on nurses” (F05, nurse, over 30) “I think that this document that came out was presented as a document that all staff could fill out, doctors, allied health, nurses, but what happens is that you get on the floor and the nurses are the ones that end up being responsible for documentation. It ends up not actually being a tool of multidisciplinary care of comprehensive care, it ends up being a tool that nurses have to fill out when they’ve got a few minutes.” (F07, nurse, 3–10) “It may be just for the clinicians not to know that all this information is coming from above and that they need to do it just in this way. They need to be part of it just to have a discussion and let them just discuss about their opinions.” (F23, nurse, 3–10)
	Access to knowledge & information *n* = 5	Lack of education and training	“There’s certainly wasn’t, In my experience, any specific training or anything like that, which I think probably would have been valuable with to explain a little bit more about.” (F01, nurse, 21–30) “Not formal training. It was more just picking up information that had been disseminated in these internal communication emails and intranet. No, it wasn’t formal, it was self-driven.” (F04, allied health, 3–10)
Individual	Opportunity *n* = 18	Lack of availability	“I think that probably in terms of implementation, I think that time constraints are probably one of the biggest factors to be mindful of.” (F07, nurse, 3–10) “There’s not a lot of time ever allocated to that, strictly speaking.” (F9, nurse, 21–30) “At the moment, we’re not allocating off the floor time, which means there’s no time for nurses to do quality improvement projects, there’s not time for training. Even just getting staff released off the floor to come to a meeting is very difficult with the busy-ness at the moment, that’s probably the biggest impact.” (F18, nurse, 11–20)
Implementation	Doing *n* = 13	Disconnect from above and staff on the floor Disconnect between the CCS and actual care	“How to make it work on the floor. I’ve lost my-- and there’s a real disconnect with that because we’ve got people in offices dictating how nurses work on the floor, but have no idea what it’s to be on the floor, and everything is on nurses….there’s a real disconnect from between the standards and the actual people on the floor.” (F05, nurse, over 30) “More filtered from the top. Yes, definitely. Not so much consultation, it was like, ’The top have made this decision, this is what you’re going to be doing. Now it’s up to you to do it‘ kind of thing.” (F22, nurse, 21–30) “A disconnect between actual care and the standard itself is people are doing it, but it’s not really so much linked to the document per se.” (F14, nurse, 11–20)
	Planning *n* = 12	The implementation plan lacked visibility and updates	“It’s certainly shared up to exec. I’m not sure about as an overall plan down to consumer level.” (F03, doctor, 21–30) “In my department, we probably don’t have like a-- How would you say? A guideline or a policy but we have it sitting up on the board and try and remind people about it.” (F10, nurse, 3–10)
	Assessing needs *n* = 6	Needs of staff were neglected	“Nursing Uh has very much become a this is what we’re doing and this is how you do it and just do it rather than really asking how we feel about it.” (F01, nurse, 21–30) “I think it’s not only been labor intensive but I think there is a little bit of bitterness towards it as well because we don’t feel like we’ve been consulted, and then the document itself feels just a bit disrespectful I think to our ability as nurses as well.” (F07, nurse, 3–10)
	Teaming *n* = 6	Soloing and poor cross communication	“Sometimes the communication upwards or downwards from and to the committee in a big hospital it’s hard to get all the information into one room at the one time” (F03, doctor, 21–30) “There is room on this comprehensive care plan for written communication between teams, but that doesn’t really happen. I don’t know that anyone other than the nurses look at this comprehensive care even though everyone is able to document on it and write notes on it. I think it’s unfortunate that they’re encouraging written communication because I just don’t think that is nearly as effective as verbal communication.” (F07, nurse, 3–10) “Because they’ve had a couple of teams and one saying one thing and one saying another in their eyes. Whereas we might know what’s going on, it’s not communicated very well to the patients.” (F15, nurse, over 30) “Compartmentalised or siloed…. So it seems to be fairly decent in the individual sections, but not a lot of crosstalk.” (M02, allied health, 3–10)

Note. The term ‘consumers’ refers broadly to patients, families, and carers. CCS: Comprehensive Care Standard. CNC: Clinical Nurse Consultant.

**Table 3:** Enablers to the Implementation of the Comprehensive Care Standard coded using the Consolidated Framework for Implementation Research (CFIR).

CFIR Domain	Construct	Enabler Statement	Exemplar Quote (ID, Role, Years of Work Experience)
Innovation	Relative Advantage *n* = 8	Implementing CCS is better than current practice	“It’s creating the most targeted approach towards having a clear goal with standardised language and standardised planning around patient care. It does create continuity and better actionable plans….” (F04, allied health, 3–10) “I think it’s actually better because we didn’t have certain things before and now we do. Say for example, behavioural management and what was the other one? Something suicide that used to be ED-focused. We don’t look at that in an inpatient perspective. But because of the standard, then every single clinician has to learn to identify that as part of their care to the patient.” (F11, nurse, 11–20)
Outer setting	Partnerships & Connections *n* = 17	Hospital is networked with external entities	“And in terms of working with [local HHS], they actually decided on the focus month of the standards per month. It’s come from them and they’re the ones that set out the audit tools based on the standards and we report to them basically. And then they report back on the audit results asking for action plans.” (F11, nurse, 11–20) “We’re all linked, obviously. [MyHospital] has a close connection with [Hospital1] and the [Hospital2] and [Hospital3], and there’s systems in place where we can talk. We talk on Teams, we talk up.” (F15, nurse, over 30) “There’s quite a few statewide committees. …the Standard 5, we’ve got statewide groups for care plan risk assessment, falls, pressure injuries. They’re the three main ones. Then we have a statewide coordinator, who arranges all of the stuff for Standard 5, who’s just that one go-to person that’s that center of resources. We can all see each other’s forms and policies, so that we have that information-sharing by benchmarking with each other constantly.” (F18, nurse, 11–20)
Inner setting	Access to knowledge & information *n* = 23	Education, training and information are assessable	“There was an education plan put in place, and then that was implemented through the whole hospital, and then they had a go live date. The education happened prior to the go live date. … There was the policy they did a pre-recorded webinar thing that was implemented, so staff could watch that at any time. They had some quick reference guides that our IT people did, which is a fact sheet sort of thing. There was a list of frequently asked questions that is on the internet that can be accessed at any time. Then there was lots of in-person face-to-face sessions that were run by the champions of the ward and the hospital. ....” (F06, nurse, 11–20) “They also send out a regular fact sheet for highlighting the different standards to constantly have it on our radar in a way and with a little quiz around the standard.” (F10, nurse, over 30) “So this real time bedside education, … And basically, we upskill the team leaders. And when there’s a new admission, the facilitator will work with that nurse and do it real time, real questions with the patients as opposed to education, like at 2:30 sit in the classroom and being told, ‘Hey, you should do it this way’. So it is just a different way of doing it and really doing it real time.” (F11, nurse, 11–20)
	Structural characteristics *n* = 19 Information technology infrastructure *n* = 13 Leadership structure *n* = 13	Integrated documentation system and use of telehealth Having effective leadership structure to implement CCS	“And this is why I like the ieMR, is that anyone can input into that care plan. …. Once it’s activated, anyone can go in there and add things and modify things. … it’s all integrated.” (F11, nurse, 11–20) “We also then had a telehealth with that was up on the emergency board with the [HospitalName1] trauma team.” (F01, nurse, 21–30) “We now have an integrated viewer system so that a lot of people can see clinician’s records, documentations, and care provision, care continuity, avoid service duplication, new referrals; all those kinds of things. So, yeah, that is ongoing and in the digital in the ieMR world, service integration, you know, systems used to be in different steps, and now they are all integrated into a package so one talks to the other” (M04, nurse, over 30)
	Available resources *n* = 19	Resources are available, e.g., risk screening and assessment tools and protocols	“Through those positions, we received resources such as Excel templates to track progress on actions that we were trying” (F16, allied health, 3–10) “We have good processes, we have the right documentation, procedures, care plans, checklists, toolkits, audit tools” (M01, nurse, 21–30)
	Communication *n* = 9	Good communication among staff and consumers, e.g., MDT meeting that involves consumer	“I think the areas in which the multidisciplinary care works is when you have got that really strong communication, which often happens through those multidisciplinary meetings where you have a physio, you have an OT, you have the doctors and a senior nurse, and you just run through every single patient and go, “Okay, what’s everyone’s priority? Where’s everybody at? We’ve cleared them all right, let’s get them home,” and things like that.” (F7, nurse, 3–10) “I’m part of an MDT team and we’re very good at communicating with each other if somebody is needing a service and referral…[what’s app] it’s a very good tool. These registrars and surgeons doing meetings and discussing cases, you can-- I’m sure you can contact them, and they do respond. They’re on the ball.” (F21, doctor, 21–30)
	Compatibility *n* = 7	The CCS fits current workflow, system or processes	“We had already been starting to put a little bit more focus on doing regular frailties at and frailty scores from ED before implementation. I think that did formalise those things a little bit more.” (F03, doctor, 21–30) “It does actually come up quite a lot that it was like we’ve already been doing this, but now we have a name for all the things that we’re doing. Seems to be very, very commonly comes up. It’s now we can actually point to something on the wall that describes what we actually have just been doing naturally without really putting it into terms.” (F20, nurse, over 30)
	Culture *n* = 6	Good working culture, supportive and encouraging, sharing knowledge, person-centred	“We have a really good working culture, …. Everyone involves talks to each other and involves the family … It’s a very good work workplace culture to work in, very supportive and encouraging and that’s from all staff, everyone is like that.” (F13, allied health, less than 3) “We always endeavor for good family-centered care. …I do firmly believe that a culture of people working together and people sharing knowledge and people being willing to help and share their experiences where good comprehensive care comes from. Sometimes you do worry that with such high turnover of staff, with staff feeling junior. I think the best comprehensive care comes from good old, robust nurses that you want to be in there and doing it.” (F14, nurse, 11–20)
Individual	Implementation leads *n* = 19	Having implementation leads that drive the CCS implementation	“We have a clinical nurse consultant for Comprehensive Care. She has been integral in-- I suppose you can say developing it and leading it….Everything she does is reported up through executive, through the committee, and then it comes back down, any information needed.” (F20, nurse, over 30) “We create what we call a Standard Lead, so if someone is the Standard Lead, that person then has got a group of people who are champions.” (M04, nurse, over 30)
Implementation	Engaging Innovation recipients *n* = 22	Engaging consumers in implementation and in care planning and delivery	“We certainly always include patients and their families in care. Most probably anything that we’re doing in relation to the comprehensive care standard is most probably also wrapped up in the communicating for safety in standard success and with bedside handover and we talk about, when we do it at bedside, what we’re doing to pressure injury prevention, managing falls, that type of stuff around it. They’re also given information regarding pressure injury prevention and falls and all of that type of stuff part of their admission packs. … All our patients have TVs in their room, so if they’re not watching anything, all that comes up in through as well.” (F05, nurse, over 30) “We have a consumer advisory representative that’s usually on most of the committee level, they can provide feedback on procedural documents, guidelines, approaches to whatever we’re trying to achieve.” (F16, allied health, 3–10)
	Reflecting & evaluating Implementation *n* = 21	Collect and discuss feedback from both consumer and staff	“We do post-surveys. There’s one, I think, every quarter. Then they also do the PREMs, the phone, the text one so they do.” (F05, nurse, over 30) “I think it was just sitting down with the nurses and saying what they thought, what went right, what didn’t go right, what could be improved.” (F06, nurse, 11–20)
	Planning *n* = 14	Implementation plan was in place	“We have our overarching documents in terms of strategic plans, clinical plans, health equity plans and I guess it’s built into all of those strategic documents and the implementation plans that come from that.” (F16, allied health, 3–10) “Comprehensive Care Committee meets monthly have an action plan where we review quality initiatives, our audit evaluation work collaboratively to ensure successful implementation at scale for the hospital.” (M01, nurse, 21–30)
	Teaming *n* = 10	Working with multidisciplinary teams collaboratively	“It’s very good at [MyHospitalName]l, the multidisciplinary care… There was a good amount of teamwork involved with delivery of the baby and then getting the baby to neo-resus.” (F02, nurse, 3–10) “Most patients in the hospital, though, are discussed at a multidisciplinary meeting, and sometimes for some units, that’s every day for our quick turnover units. We have one once a week, and that’s really where we actually sit down and talk about the plans for patients, where they’re going.” (F08, nurse, over 30)

Note. The term ‘consumers’ refers broadly to patients, families, and carers. CCS: Comprehensive Care Standard; MDT: multidisciplinary teams, ieMR: integrated electronic medical record. PREMs: Patient Reported Experience Measures.

**Table 4:** Summary of Consolidated Framework for Implementation Research (CFIR) barrier and enabler constructs mapped to Expert Recommendations for Implementing Change (ERIC) strategies.

CFIR Construct	Barrier	Enabler	ERIC Strategy (Most Strongly Recommended)	% of Agreement
Complexity	√		Develop a formal implementation blueprint Promote adaptability	43 40
Adaptability	√		Promote adaptability	73
Assessing needs	√		Obtain and use staff feedback Involve staff	76 71
Structural characteristics	√	√	Assess for readiness and identify barriers and facilitators	36
Available resources	√	√	Access new funding	78
Access to knowledge & information	√	√	Conduct educational meeting	79
Culture	√	√	Identify and prepare champions	52
Doing	√	√	Purposely reexamine the implementation	45
Planning	√	√	Develop a formal implementation blueprint	73
Reflecting & evaluating implementation		√	Develop and implement tools for quality monitoring Audit and provide feedback	60 56
Implementation leads		√	Identify and prepare champions	64
Engaging Innovation recipients		√	Involve patients and family members	59
Communication		√	Promote network weaving Organise clinician implementation team meetings	57 52
Relative Advantage		√	Identify and prepare champions	45
Partnerships & Connections		√	Build a coalition	62
Compatibility		√	Promote adaptability Conduct local consensus discussions	45 41

Note: Critical Incidents, Opportunity, and Teaming do not currently have mapped strategies in the CFIR-ERIC Matching Tool.

## Data Availability

The datasets generated and/or analysed during the current study are not publicly available due to privacy or ethical restrictions but are available from the corresponding author on reasonable request.

## Supplementary Materials

The following supporting information can be downloaded at: https://www.mdpi.com/article/10.3390/nursrep15120428/s1, File S1: Staff interview guide; File S2: codebook; File S3: COREQ checklist; File S4: Implementation enhancement strategies.

## Public Involvement Statement

From inception, we partnered with members of the eQC (evaluating Quality of Care) Patient and Carer Advisory Board for their input and feedback regarding the protocol, analysis, and results by presenting and discussing the research regularly at board meetings.

## Guidelines and Standards Statement

This manuscript was drafted against the Consolidated Criteria for Reporting Qualitative Studies.

## Abbreviations

The following abbreviations are used in this manuscript:

ACSQHC	Australian Commission on Safety and Quality in Health Care
CCS	Comprehensive Care Standard
CFIR	Consolidated Framework for Implementation Research
CNC	Clinical Nurse Consultant
eQC	Evaluating Quality of Care
ERIC	Expert Recommendations for Implementing Change
ieMR	Integrated Electronic Medical Record
MDT	Multidisciplinary Team
NSQHS	National Safety and Quality Health Service
PREMs	Patient Reported Experience Measures
