# Supplementary material for: Barriers, Enablers, and Impacts of Implementing National Comprehensive Care Standards in Acute Care Hospitals: An Interview Study
Source: Nurs Rep. 2025 Dec 2;15(12):428. doi: 10.3390/nursrep15120428 (PMC12735530; doi:10.3390/nursrep15120428)
Supplement: Supplementary file 1 [file nursrep-15-00428-s001.zip › Supplementary file 1. Staff Interview guide.pdf]

Evaluating Comprehensive Care (eCC) project Staff Interview Guide

#	Constructs	Target[i]	Questions	Y/N
**A.**	I'll ask you a few questions about the communication strategy and people involved in the planning stage of implementing the Comprehensive Care Standard.			
1	Process- Engaging: Key Stakeholders		1. Communication strategy<br>(1) How did you first hear about the Comprehensive care standard?<br>(2) What is the communication strategy for getting the word out about (implementing the) Comprehensive Care Standard at your organisation?<br>(3) What materials/modes/venues were used? e.g., e-bulletin boards, emails, brochures?<br>(4) What process were used to communicate? e.g., going to staff meetings, talking to people informally?<br>(5) How effective do you think they are?	
2	Individual-Knowledge & Beliefs about the Intervention; Intervention-Evidence Strength & Quality/Design Quality & Packaging; Inner setting-Relative Priority		2. Perception of the Standard<br>(1) how do you feel about the comprehensive care standard?<br>a. Is it complex or simple? is it too vague or too specific?<br>b. Is it evidence based? Is it helpful? is it adaptable to your local context?<br>(2) How does the Comprehensive Care Standard address the priorities and objectives in your setting?<br>(3) What are issues that have arisen from the implementing the Comprehensive Care Standard? e.g., staff burnout/complaints, Increased workload?	
3	Inner setting-readiness for implementation: leadership engagement; Process-Engaging: Opinion Leaders/ Formally Appointed Internal Implementation Leaders		3. Leadership<br>(1) Who have been involved in leading the implementation of the Comprehensive Care Standard in your department? Instead of using names, you can just say the work title or position of the person.<br>(2) How did they come into this role? e.g. Appointed? Volunteered? Told to volunteer?<br>(3) Do they have availability, capability, and power to fulfill this leadership role?<br>(4) How well do you think they lead the implementation? Is leadership lacking?	
4	Individual-implementation facilitators/ implementation leads		4. Champions<br>(1) Other than the formal implementation leader, are there people who facilitate or championed[ii] (go above and beyond what might be expected) the implementation of the Comprehensive Care Standard?<br>(2) Were they formally appointed in this position, or was it an informal role?<br>(3) How do you think they helped with implementation?	
5	Individual-deliver/ recipient; Process-engaging	Imp.⇒	5.Key Stakeholders<br>(1) Who were the key stakeholders[iii] involved in implementing the Comprehensive Care Standard?<br>(2) What steps have been taken to encourage Key Stakeholders to help with implementation?<br>(3) How frequently and how were they communicated?	
6	Outer setting-Patient Needs & Resources; Inner setting: Tension for Change; Process-Engaging: Key Stakeholders/ Intervention Participants		6. Needs of consumers<br>(1) To what extent do you think the needs and preferences of the patients were considered when planning to implement the Comprehensive Care Standard?<br>(2) How well do you think implementing the Standard meets the needs of the consumers?	

#	Constructs	Target[i]	Questions	Y/N
7	Characteristics - needs		7. Needs of staff<br>(1) To what extent do you think were the needs and preferences of carer professionals (i.e. doctors, nurses, allied health) considered when planning to implement the Comprehensive Care Standard? e.g., well-being and personal fulfillment; awareness, knowledge and skills.<br>(2) How well do you think implementing the Standard meets their needs?	
**B.** Here are some questions related to the implementation plan of the Comprehensive Care Standard.				
8	Intervention Source; Process-planning; Process- Engaging: Key Stakeholders; Intervention-Complexity;	Imp.	8. Implementation Plan<br>(1) Is there a plan for implementing the Comprehensive Care Standard in your setting? [If yes.]<br>(2) Can you describe the plan?<br>(3) How detailed is the plan? Is the plan overly complex? Understandable? Realistic and feasible?<br>(4) What was done to get a plan in place to implement the Comprehensive Care Standard in your setting?	
9	Process-planning; Process-Executing	Imp.	9. Execution<br>(1) What role has the implementation plan played during implementation? e.g. Used to guide implementation of the Comprehensive Care Standard? Used to compare planned with actual progress?<br>(2) Were there revisions or refinements to the plan? due to COVID? Other barriers, mistakes?<br>(3) Was the plan shared/reviewed with other stakeholders? If so, how regularly?	
**C.** Here are some questions related to the changes to make the implementation happen.				
10	Intervention-Adaptability; Intervention-Cost	Imp.⇒	10. Change<br>(1) What changes or alterations have been done to support implementing the Comprehensive Care Standard? [if yes] Can you tell me more about it? Changes include:<br>a. Scope of practice? Priorities for clinicians/organisations?<br>b. Changes in formal policies? Workplace flow/processes?<br>c. Job descriptions or staff ratio?<br>(2) Do you feel any changes could have been done to support the implementation?<br>(3) What costs do you think were incurred to implement the Comprehensive Care Standard? *Are they affordable?*	
11	Inner setting-Structural Characteristics	Care	11. Infrastructure<br>(1) We talked about changes in policies and practices, but what changes have been done in information technology (IT) systems or electronic records systems?<br>(2) Do you feel any changes could have been done to support the implementation?	
**D.** Here are some questions related to the Compatibility of the Comprehensive Care Standard.				
12	Inner setting- Implementation Climate: Tension for Change/ Compatibility/ relative priority; Inner setting- Readiness for Implementation:		12. Previous work<br>(1) How do you feel about previous programs/practices/processes that were available related to the implementing the Comprehensive Care Standard?<br>(2) How does implementing the Comprehensive Care Standard fill previous gaps in patient care?	
13	Knowledge & Beliefs about the Intervention		13. Existing work<br>(1) How well does the intervention fit with existing work processes and practices in your setting?<br>(2) What are issues that have arisen from the implementing the Comprehensive Care Standard? e.g., staff burnout/complaints, Increased workload?	

#	Constructs	Target[i]	Questions	Y/N
14			14. Priorities and objectives<br>(1) How does the implementing the Comprehensive Care Standard address the priorities and objectives in your setting?<br>(2) how important do you think it is to implement the comprehensive care standard compared to the other priorities?	
**E.** Let us talk about patient care.				
15	Intervention-specific Qs; Process-engaging		15. Patient care plan<br>(1) Do patients served at your hospital have a care plan? [if yes] Can you tell me more about it?<br>(2) Is it a standardised overarching care plan template used across the whole hospital?<br>(3) when does the development of a comprehensive care plan start during an episode of care? how current does the plan remain?<br>(4) who are involved in making a comprehensive care plan? Are patient and families involved?<br>(5) what content is included in it? whether the patients' goals of admission were ever documented?<br>(6) how is the plan used in daily care? Is the plan shared to GP at discharge?<br>(7) did the plan increase patient driven goals and relevant outcome reporting?<br>(8) is there a care coordinator of the plan and how did the person come into this role? e.g. Appointed?<br>(9) whether this plan facilitates streamlined documentation and communication?	
**F.** Let us talk about consumers.				
16	Process-Engaging: Intervention Participants		16. Consumer engagement<br>(1) How are patients and their family and carers involved in their care? e.g. making a care plan<br>(2) How about patient aged 65 years or over? Or patients with CI? Are there any differences in engagement?	
17	Outer setting-Patient Needs & Resources		17. Barriers and facilitators for consumers<br>(1) What barriers do patients and their families face to be involved in patient-centred care? e.g. language barriers, poor health literacy, awareness?<br>(2) What facilitators do patients and their families face to be involved in patient-centred care? e.g. patient education? Information booklet?<br>(3) Any different/new barriers or facilitators during the COVID pandemic?<br>(4) what do you think can be done to improve patient engagement in their care?	
18	Intervention-specific Qs; Outer setting-Patient Needs & Resources; Process-Engaging: Key Stakeholders/ Intervention Participants		18. Outcome<br>(1) Were there any changes in the care they received after implementing the Comprehensive Care Standard? e.g. More screening and assessments? More shared decision making? More multidisciplinary care? Improved access to services?<br>(2) Did implementing the Comprehensive Care standard bring any changes in patient outcomes? e.g. adverse events, quality of life	
19			19. Consumer feedback<br>(1) Have your hospital/department elicited information from patients regarding their experiences with the care they received?<br>(2) What are their perceptions of the care they received?<br>(3) Can you describe what kind of specific information you have heard?	

#	Constructs	Target[i]	Questions	Y/N
**G.** Here are some questions about your workplace culture.				
20	Inner setting- Culture; Individual- Individual Identification with Hospital		20. Workplace culture<br>(1) How would you describe the culture[iv] of your hospital?<br>(2) To what extent does your hospital prioritize continuous learning to support ongoing quality improvement efforts? e.g. use of data or other evidence to inform change<br>(3) Do doctors, nurses, and allied health professionals in your hospital have equal accountability in relation to implementing the Comprehensive Care Standard?<br>(4) How do you think your hospital's culture[v] has affected the implementation of the Comprehensive Care Standard?	
21	Outer setting-Cosmopolitanism; Process: External Change Agents	Imp. ⇒	21.1 Network & External help<br>(1) How extensively is your hospital networked with external partners, such as other hospitals, research facilities, local health authorities, in relation to implementing the Comprehensive Care Standard?<br>(2) Who? How? Were they helpful?<br>21.2 Network & External help<br>(1) How extensively does your department collaborate with other departments within the hospital to implement the Comprehensive Care Standard in your setting?	
22	Outer setting- Pressure	Imp.	22.1 Pressure<br>(1) Other hospitals are implementing the Comprehensive Care Standard as well. Does your hospital/department have any pressure that drive implementation and/or delivery of the comprehensive care standard? e.g. accreditation assessment, community, Local health authority.<br>22.2 Pressure<br>(1) Other units within your hospital implementing the Comprehensive Care Standard as well. Do you feel any pressure that drive implementation or delivery of the comprehensive care standard?	
23	Inner setting- Implementation Climate: Hospital Incentives & Rewards; Implementation Climate: Learning Climate	Imp. ⇒	23. Incentives; Acknowledge of Involvement<br>(1) What kinds of incentives (e.g. recognitions or reward) are there to help ensure that the implementation of the Comprehensive Care Standard is successful in your hospital?<br>(2) What is your motivation for wanting to help ensure the implementation is successful?<br>(3) how is your involvement in implementing the Comprehensive Care Standard valued and recognized?	
**H.** Here are some questions about the Reflecting & Evaluating of the implementation				
24	Process- Reflecting & Evaluating	Imp.	24. Accreditation<br>The Standard was released by the Commission (Australian Commission on Safety and Quality in Health Care) in 2017 and starting from 2019 all hospital were required to be assessed to obtain accreditation.<br>(1) are you aware of your hospital's accreditation assessment and results?<br>(2) Were there any issues regarding the results?<br>(3) Did your hospital struggle to obtain accreditation?	

#	Constructs	Target[i]	Questions	Y/N
25	Inner setting- Implementation Climate: Goals and Feedback; Process- Reflecting & Evaluating	Imp.	25. Feedback about implementation<br>(1) We talked about feedback from consumers, is there feedback about the implementation of the Comprehensive Care Standard itself from other sources? e.g., staff members, peer reviews, performance reviews. [if yes]<br>(2) How often do you get them? Where do they come from? Is feedback elicited from staff? Consumers?<br>(3) What do they look like? Content, mode, form? Who designed them?<br>(4) How helpful do you think they are? How could they be improved?<br>[if no] what feedback do you think will be helpful?	
26			26. Feedback about work<br>(1) Do you get any feedback reports about your work?<br>(2) How often do you get them?<br>(3) Where do they come from?<br>(4) What do they look like? Content, mode, form?<br>(5) How helpful are those reports? How can they be improved?	
**I.** Here are some questions related to your perceptions and experiences of the implementation of the Comprehensive Care Standard.				
27	Individual- Knowledge & Beliefs about the Intervention/ Self-efficacy; Intervention- Evidence Strength & Quality/Design Quality & Packaging		27.Belief<br>(1) How do you feel about the implementation of the Comprehensive Care Standard in your hospital? is it effective?<br>(2) Do you know any kind of supporting evidence or proof about the effectiveness of the implementation?<br>(3) What is your perception of the quality of the supporting materials, packaging, and bundling of the intervention for implementation? Why?	
28	Individual-Self-efficacy		28. Confidence<br>(1) How confident are you that the Comprehensive Care Standard has been successfully implemented at your hospital? On a scale of 0-10?<br>(2) What gives you that level of confidence (or lack of confidence)?	
29	Inner setting- Readiness for Implementation: Available Resources/ Access to Knowledge & Information	Imp.	29.1 Resources<br>(1) What kind of support and resources did you receive to implement and administer the Comprehensive Care Standard?<br>(2) What do you feel was lacking?	
		Care	29.2 Resources<br>(1) What kind of training and information did you receive to perform comprehensive care?<br>(2) What do you feel was lacking?	
30	Inner setting- Networks & Communications	Care	30. Work relationships<br>(1) Can you tell me a story about a time you needed to work with others to develop and deliver a patient care plan? either successful or unsuccessful story.	

Note: [1] "Imp." Is targeted at people who are involved in implementing the Comprehensive Care Standard only. "Care" is targeted at people who are involved in care delivery only. [2] Champions: Individuals who dedicate themselves to supporting, marketing, and 'driving through' an implementation, overcoming indifference or resistance that the intervention may provoke in a hospital. [3] key stakeholders: an individual or group who has a significant interest or concern in the care standard, and whose actions or decisions can have an impact on the outcome or success of the implementation, such as (1) patients, family members, caregivers; (2) doctors, nurses, allied health professionals, social workers; (3)

hospital management, regulatory agencies (state and federal government bodies). [4] Culture: general beliefs, values, and assumptions that people embrace. [5] Culture: general beliefs, values, and assumptions that people embrace.
