# Supplementary material for: Barriers, Enablers, and Impacts of Implementing National Comprehensive Care Standards in Acute Care Hospitals: An Interview Study
Source: Nurs Rep. 2025 Dec 2;15(12):428. doi: 10.3390/nursrep15120428 (PMC12735530; doi:10.3390/nursrep15120428)
Supplement: Supplementary file 1 [file nursrep-15-00428-s001.zip › Supplementary file 2. Codebook.pdf]

Codebook

Name	Description
1. Communication strategy about CCS	
2. Perception of the implementation	
3. Perception of the standard	
advantage	
disadvantage	
4. Implementation people	
facilitator	Include champion
recipient	Related to needs of consumer
deliverer	
leadership	
leader	
lead	
other key stakeholder	
team structure	
5. Needs of staff	
6. Implementation Plan	
7. Doing or Execution	
8. Change	
cost, funding	
policy or protocol	

Name	Description
flow or processes	
practices	
scope or priority	
staff ratio, job description	
tools	
documentation system	
9. Infrastructure	
IT Infrastructure - documentation system	
work infrastructure	
10. Outcome, impact, efficacy	
access to health services	
patient care	
health outcome	
impact on staff	
11. Patient care plan	
care planning and review	
consumer involvement	
coordinator	
goal setting and outcome report	
sharing and discharge	
start, assessment	
template	

Name	Description
use and communication	
12. Consumer engagement	Related to recipient-centred culture
13. Consumer engagement – affecting factor	
barriers	
facilitator	
14. Needs of consumer	
15. Outer setting	including ACSQHC network
covid	
rurality	
16. Feedback	
Consumer feedback	
Other feedback	Include feedback about the CCS, staff, feedback from peer review
17. Workplace culture	
18. Reporting culture	
19. Network & External help	
20. External & Peer Pressure	
21. Incentives; Acknowledge of Involvement	
22. Reflecting & Evaluating	
23. Teamwork and communication	
24. Resources	
25. Education, training, information, culture	Related to learning-centred culture
Other	

Name	Description
