# Supplementary material for: Barriers, Enablers, and Impacts of Implementing National Comprehensive Care Standards in Acute Care Hospitals: An Interview Study
Source: Nurs Rep. 2025 Dec 2;15(12):428. doi: 10.3390/nursrep15120428 (PMC12735530; doi:10.3390/nursrep15120428)
Supplement: Supplementary file 1 [file nursrep-15-00428-s001.zip › Supplementary file 3. COREQ checklist.pdf]

Consolidated criteria for reporting qualitative studies (COREQ): 32-item checklist

Developed from:
Tong A, Sainsbury P, Craig J. Consolidated criteria for reporting qualitative research (COREQ): a 32-item checklist for interviews and focus groups. *International Journal for Quality in Health Care*. 2007. Volume 19, Number 6: pp. 349 – 357

**No. Item**	**Guide questions/description**	**Reported on section**
**Domain 1: Research team and reflexivity**		
Personal Characteristics		
1. Interviewer/facilitator	Which author/s conducted the interview or focus group?	4.7 Research team and reflexivity
2. Credentials	What were the researcher's credentials? E.g. PhD, MD	4.7 Research team and reflexivity
3. Occupation	What was their occupation at the time of the study?	4.7 Research team and reflexivity
4. Gender	Was the researcher male or female?	4.7 Research team and reflexivity
5. Experience and training	What experience or training did the researcher have?	4.7 Research team and reflexivity
Relationship with participants		
6. Relationship established	Was a relationship established prior to study commencement?	4.7 Research team and reflexivity
7. Participant knowledge of the interviewer	What did the participants know about the researcher? e.g. personal goals, reasons for doing the research	4.7 Research team and reflexivity
8. Interviewer characteristics	What characteristics were reported about the inter viewer/facilitator? e.g. Bias, assumptions, reasons and interests in the research topic	4.7 Research team and reflexivity
**Domain 2: study design**		
Theoretical framework		
9. Methodological orientation and Theory	What methodological orientation was stated to underpin the study? e.g. grounded theory, discourse analysis, ethnography, phenomenology, content analysis	4.2 Theoretical framework
Participant selection		
10. Sampling	How were participants selected? e.g. purposive, convenience, consecutive, snowball	4.3 Study setting and recruitment
11. Method of approach	How were participants approached? e.g. face-to-face, telephone, mail, email	4.3 Study setting and recruitment
12. Sample size	How many participants were in the study?	5.1 Sample characteristics

13. Non-participation	How many people refused to participate or dropped out? Reasons?	N.A.
Setting		
14. Setting of data collection	Where was the data collected? e.g. home, clinic, workplace	4.3 Study setting and recruitment
15. Presence of non-participants	Was anyone else present besides the participants and researchers?	No
16. Description of sample	What are the important characteristics of the sample? e.g. demographic data, date	5.1 Sample characteristics
Data collection		
17. Interview guide	Were questions, prompts, guides provided by the authors? Was it pilot tested?	4.5 Data collection
18. Repeat interviews	Were repeat inter views carried out? If yes, how many?	No
19. Audio/visual recording	Did the research use audio or visual recording to collect the data?	4.6 Data analysis
20. Field notes	Were field notes made during and/or after the inter view or focus group?	4.6 Data analysis
21. Duration	What was the duration of the inter views or focus group?	4.5 Data collection
22. Data saturation	Was data saturation discussed?	4.3 Study setting and recruitment
23. Transcripts returned	Were transcripts returned to participants for comment and/or correction?	4.5 Data collection
**Domain 3: analysis and findings**		
Data analysis		
24. Number of data coders	How many data coders coded the data?	4.6 Data analysis
25. Description of the coding tree	Did authors provide a description of the coding tree?	4.6 Data analysis
26. Derivation of themes	Were themes identified in advance or derived from the data?	4.6 Data analysis
27. Software	What software, if applicable, was used to manage the data?	4.6 Data analysis
28. Participant checking	Did participants provide feedback on the findings?	Not on findings, some checked the transcripts
Reporting		
29. Quotations presented	Were participant quotations presented to illustrate the themes/findings? Was each quotation identified? e.g. participant number	5. Results
30. Data and findings consistent	Was there consistency between the data presented and the findings?	5. Results & 6. Discussion
31. Clarity of major themes	Were major themes clearly presented in the findings?	5. Results & 6. Discussion
32. Clarity of minor themes	Is there a description of diverse cases or discussion of minor themes?	5. Results & 6. Discussion
